# QTL for tuber morphology traits in diploid potato

**DOI:** 10.1007/s13353-018-0433-x

**Published:** 2018-02-28

**Authors:** Agnieszka Hara-Skrzypiec, J. Śliwka, H. Jakuczun, E. Zimnoch-Guzowska

**Affiliations:** Plant Breeding and Acclimatization Institute - National Research Institute, Platanowa 19, 05-831 Młochów, Poland

**Keywords:** Mapping, Morphology, *Solanum tuberosum*, Tuber quality

## Abstract

A diploid, potato mapping population consisting of 149 individuals was assessed in three consecutive years for important agronomic and quality traits: tuber shape, regularity of tuber shape, eye depth, mean tuber weight, and tuber flesh color. Analysis of variance showed that the genotype had the largest influence on the phenotypic scores but effect of the genotype × year interactions was also strong. Using this data and an existing genetic map, a quantitative trait loci (QTL) analysis was conducted. From four to seven QTL were detected for each trait except tuber flesh color, which was determined by a major QTL on chromosome III explaining 76.8% of the trait variance. Additionally, a minor QTL for flesh color was localized on chromosome II. For the other traits, significant QTL were detected: for tuber shape on chromosome X, for regularity of tuber shape on chromosome III, for eye depth on chromosome IV, and for tuber weight on chromosome I. Some detected QTL confirmed previous studies, but new ones were also identified.

**Electronic supplementary material:**

The online version of this article (10.1007/s13353-018-0433-x) contains supplementary material, which is available to authorized users.

## Introduction

New potato cultivars should not only fulfill the strict yield and tuber quality requirements but also should be resistant to biotic and abiotic stresses. Morphological traits of potato—tuber shape, eye depth, regularity of tuber shape, and tuber weight—are considered to be essential in breeding for tuber quality. The shape of potato tubers is important for processing industry and fresh market. Cultivars with round tubers are used for crisps, while long tubers are recommended for French fries (van Eck et al. 1994). Preferred by consumers, table cultivars have oval or long-oval tubers. Previous studies on inheritance of tuber shape have shown contradictory results. Part of them has suggested it to be a monogenic trait (Taylor 1978; Okwuagwu 1981; Masson 1985; van Eck et al. 1994), while some works have identified many loci on various chromosomes responsible for trait variation (Sørensen 2006; Śliwka et al. 2008; D’hoop et al. 2014 and Prashar et al. 2014). Deep eyes decrease tuber appearance and lead to an increase of peeling cost in processing industry (Li et al. 2005). According to Li et al. (2005), this trait is determined by a locus *Eyd* on chromosome X with a dominant allele causing deep eyes. Other studies have shown more complex architecture of the trait (Śliwka et al. 2008; Prashar et al. 2014). Regularity of tuber shape is a weakly understood trait depending on the depth of indentations at the rose and the heel ends and on other deviations from ideal shape (Domański 2001). Important part of its evaluation is the depth of tuber eyes. Hitherto genetic analysis for this trait was conducted by Śliwka et al. (2008) and Bradshaw et al. (2008). Depending on the potato use, various tuber size is preferred by consumers and industry. Genetics of mean tuber weight was so far analyzed by Manrique-Carpintero et al. (2015), Bradshaw et al. (2008), and D’hoop et al. (2014). Another trait, potato tuber flesh color, is determined by the carotenoid content. Their high level has a particular value for human health. Carotenoids act as antioxidants. They are major pigments of the yellow spot in the human retina and protect it from damage (Morris et al. 2004). Tuber flesh varies from white to deep yellow. Consumers’ preferences concerning flesh color depend on the region of the world and local tradition. The yellow flesh is considered to be controlled by the single dominant allele *Y* at locus *Y* on potato chromosome III (Bonierbale et al. 1988). According to Brown et al. (1993), the presence of a dominant allele *Or* close to or at *Y* locus determined orange flesh color. This hypothesis was based on study using a hybrid population *S. phureja* × *S. stenotomum*. Wolters et al. (2010) showed that orange flesh tubers are produced only by genotypes with dominant beta-carotene hydroxylase (*Chy2*) allele in combination with homozygosity for the recessive allele of zeaxanthin epoxide (*Zep*). According to Kloosterman et al. (2010), a *beta-carotene hydroxylase* (*bch*) gene is occupying the *Y* locus on chromosome III and is the most promising candidate gene for encoding the tuber flesh color.

We have previously used Diversity Array Technology (DArT) markers to construct a linkage map of diploid mapping population 11–36 and perform mapping of QTL for susceptibility to tuber bruising and enzymatic discoloration (Hara-Skrzypiec et al. 2017). The same mapping population was investigated further in the present study to map QTL for other important potato quality traits: tuber shape, eye depth, regularity of tuber shape, flesh color, and tuber weight.

## Materials and methods

### Plant material

The mapping population 11–36 used in study (*N* = 149) is full-sibling progeny originated from cross between diploid potato clones DG 06-5 and DG 03-226. The parental forms were complex interspecific *Solanum* hybrids having in their pedigree *S. tuberosum*, *S. acaule*, *S. chacoense*, *S. demissum*, *S. gourlayi*, *S. microdontum*, *S. phureja*, *S. verrucosum*, and *S. yungasense*. In addition, DG 03-226 had a contribution of *S. stenotomum.* They resulted from a long-term recombinant breeding process at IHAR-PIB. The theoretical contributions of *S. tuberosum* in the forms DG 06-5 and DG 03-226 were 70 and 69%, respectively. The corresponding values for *S. phureja* were 6 and 14% and for *S. chacoense* were 11 and 5%. The mapping population and its parental forms were planted on 7-hill plots in two (2012, 2014) or three replications (2013) in the end of April and harvested in the end of September in three consecutive years 2012, 2013, and 2014. The plants were fertilized and treated with pesticide according to the prevailing standards.

### Phenotyping

Phenotyping of mapping population along with its parental forms was performed in three consecutive years 2012–2014. Evaluations of traits were performed in two (2012, 2014) or three (2013) 7-hill plot replications of the genotype. For the traits assessed visually, the score for a replication was the average of all tubers harvested from this replication.

*Tuber shape* was determined according to Domański (2001), who described six types of tuber shape, which were transformed into numerical scores from 1 to 6, where 1 = compressed, 2 = round, 3 = round-oval, 4 = oval, 5 = long-oval, and 6 = long.

*Regularity of tuber shape* was evaluated on a 1–9 scale, where 1 = highly malformed tubers; 2 = more than 50% of tubers malformed; 3 = 20–50% of tubers malformed and the indentations at rose and heel ends of tubers > than 4 and 3 mm, respectively; 4 = 20–50% of tubers malformed, the indentations at rose and heel ends of tubers ≤ than 4 and 3 mm, respectively; 5 = up to 20% of tubers with mild faults: protuberances among eyes, spindle- or pear-shaped tubers, the indentations at rose and heel ends of tubers ≤ than 3 and 2 mm, respectively; 6 = fairly good regularity, the indentations at rose and heel ends of tubers ≤ than 2.6 and 1.6 mm, respectively; 7 = good regularity, the indentations at rose and heel ends of tubers ≤ than 2 and 1 mm, respectively; 8 = very good regularity, the indentation at rose end of tubers ≤ than 1 mm, no indentation at the heel end; 9 = perfectly shaped tubers, no indentations at rose and heel ends. A uniformity of tuber shape was applied as an additional criterion: scores 1–6 = different types of shape may be present; 7 = most tubers of one or two types of shape; 8 = most tubers of one type of shape; 9 = almost all tubers of one type of shape. The last criterion taken into account was a ratio of largest and smallest width of tuber, which was not considered for scores 1–4, for scores 5–6 should be ≤1.6 and for scores 7–9 should be ≤1.5 (Domański 2001).

*Eye depth* was evaluated on 1–9 scale, where 1 = eyes deeper than 5 mm, 9 = eyes not perceivable by touch (Domański 2001).

*Mean tuber weight* in g was calculated as a tuber yield of 7-hill plot divided by number of tubers.

*Tuber flesh color* was evaluated visually on 5 cut tubers according to a 1–6 scale, where 1 = white, 2 = gray white, 3 = creamy white, 4 = pale yellow, 5 = yellow, and 6 = deep yellow flesh color (Domański 2001).

### Statistical analyses

The normality of distribution of phenotypic data was tested by the Shapiro-Wilk test. The reproducibility of analyzed traits between years was estimated by calculating the linear Pearson’s correlation coefficients. The determination coefficients (*R*^*2*^) for analyzed traits were estimated from an analysis of variance. The broad-sense heritability was estimated from the analysis of variance according to the formula of Domański et al. (2007): *H*^2^ = σ^2^_g_/(σ^2^_g_ + σ^2^_ge_ + σ^2^_e)_; σ^2^_g_ = (M_1_ − M_2_^)^/L; σ^2^_ge_ = M_2_ − σ^2^_e,_ where M1 = mean sum of squares effect of genotype, M2 = mean sum of squares effect of genotype × year, L = number of years.

This heritability is the ratio of genetic to total variance and differs from the heritability of progeny (clone) means averaged over years and replicates, which will be higher. The error variance has been divided by appropriate number of replicates in each year and averaged over years to give an overall error variance. All statistical analyses, histograms, and the assessment of the normality of distribution curves were done using STATISTICA for Windows (Stat Soft, Inc. and StatSoft Polska Ltd., Kraków, Polska).

### Genetic mapping and QTL analysis

Genotyping of mapping population using DArT, CAPS, and SCAR markers was performed according Hara-Skrzypiec et al. (2017). The JoinMap®4 software was used for the linkage analyses (Van Ooijen 2006). The following settings were applied: CP population type, independence LOD as a grouping parameter (linkages with LOD > 3 were considered significant), and regression mapping algorithm and Haldane’s mapping function (Śliwka et al. 2012a).The linkage groups obtained were oriented and named by comparison with maps from previous DArT mapping studies in *Solanum* (Śliwka et al. 2012a, 2012b). QTL analysis was done using interval mapping with software MapQTL®6 (Van Ooijen 2009). Detection of QTLs was done using LOD threshold > 3.0.

## Results

### Phenotyping

Mean values of 3-year evaluation of tuber shape, eye depth, regularity of tuber shape, tuber weight, and flesh color scores of the parents and individuals from the mapping population 11–36 are presented in Table 1.

**Table 1:** Mean scores of 3-year evaluation for tuber shape, regularity of tuber shape, eye depth, tuber weight, and tuber flesh color (± SD) of the parents and in the mapping population 11–36

Trait/scale	DG 03–226 ♂	DG 06–5 ♀	Mid-parent value	Population mean	Range of individual scores	Range of Pearson’s *r* values between tested years^a^
Tuber shape (1–6)	3.2 (± 0.3)	4.0 (± 0.0)	3.6	2.9 (± 0.9)	1.0–5.2	0.53–0.66
Regularity (1–9)	6.1 (± 0.1)	5.3 (± 0.2)	5.7	5.5 (± 0.4)	4.1–6.5	0.53–0.64
Eye depth (1–9)	5.6 (± 0.3)	5.0 (± 0.3)	5.3	5.4 (± 0.4)	4.3–6.7	0.60–0.65
Tuber weight (g)	45.1 (± 8.0)	24.8 (± 4.2)	30.1	35.4 (± 11.0)	14.7–65.7	0.59–0.68
Flesh color (1–6)	4.7 (± 0.3)	5.5 (± 0.5)	5.1	4.3 (± 1.0)	1.8–5.8	0.70–0.78

^a^Pearson’s correlation coefficients between the results of assessment from particular years of all tested traits were significant at α < 0.0001

**Fig. 1:**
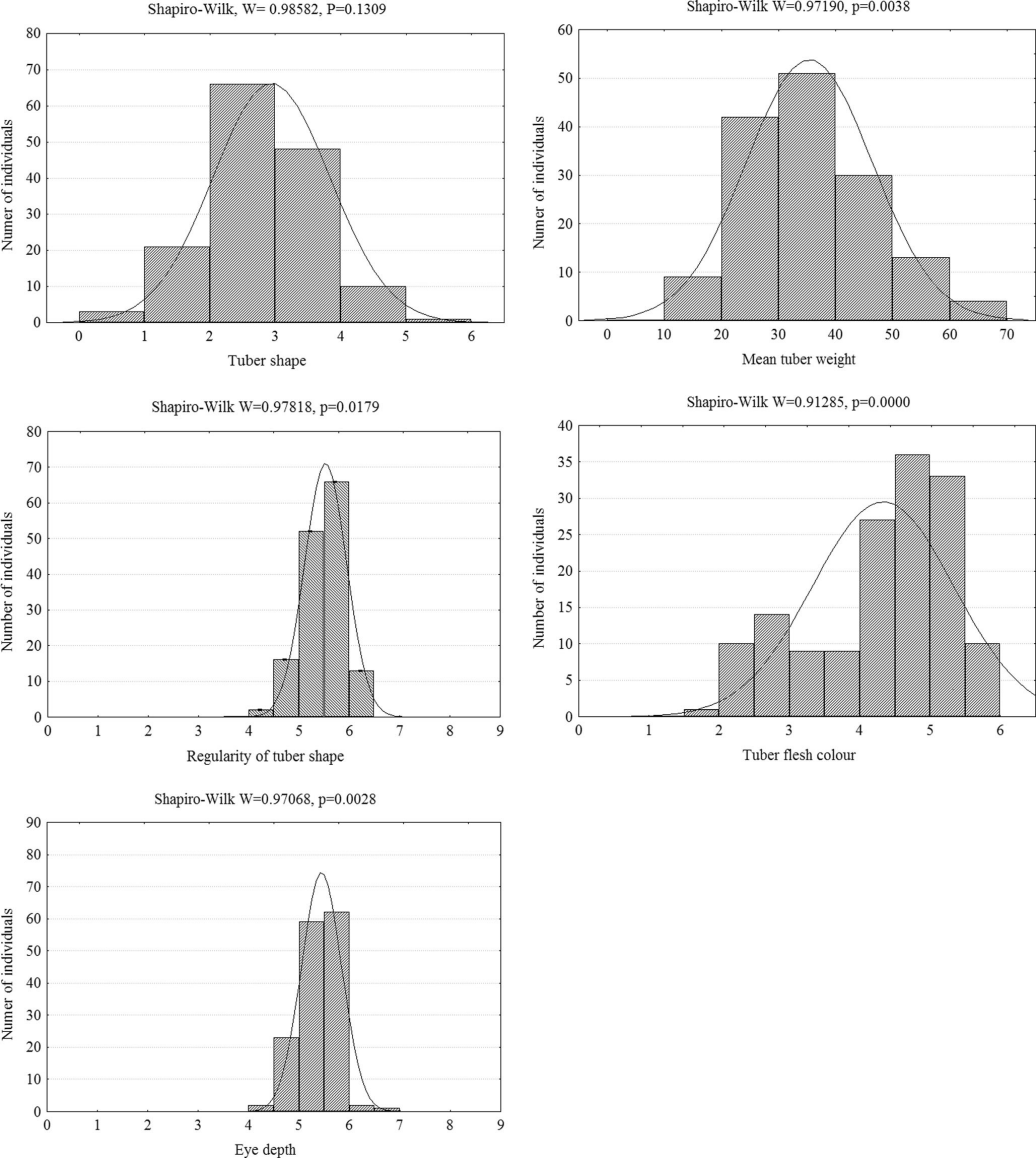
Distribution of mean (2012–2014) tuber shape (in scale 1 to 6, where 1 = compressed, 2 = round, 3 = round-oval, 4 = oval, 5 = long-oval, 6 = long), regularity of tuber shape (in 1–9 scale, where 1 = highly malformed tubers, 9 = perfectly shaped tubers), eye depth (in scale 1–9, where 1 = eyes deeper than 5 mm, 9 = eyes not perceivable by touch), tuber weight (in g), and tuber flesh color (in scale 1–6, where 1 = white, 2 = gray white, 3 = creamy white, 4 = pale yellow, 5 = yellow, 6 = deep yellow flesh color) in the mapping population 11–36. The normality of distribution of phenotypic data was tested by the Shapiro-Wilk test

Normal distribution in the progeny for tuber shape was confirmed by the Shapiro-Wilk test. Distributions for regularity of tuber shape, eye depth, tuber weight, and flesh color deviated significantly from normality. There was a great variability in the tuber shape of the progeny, covering the whole 1–6 scale and in the flesh color (1.5–6), while the ranges of progeny scores were narrowed to 4–6.5 and 4–7 for regularity of tuber shape and eye depth, respectively (Fig. 1).

Analysis of variance in the mapping population demonstrated significant effects of genotype, year, and interaction genotype × year on tuber shape, eye depth, regularity of tuber shape, tuber weight, and flesh color (Table 2). Genotype had the largest influence on tuber shape, regularity, eye depth, tuber weight, and flesh color explaining 72.6, 54.1, 55.0, 54.7, and 60.4% of the variance, respectively. The effect of the genotype × year interactions ranged from 18.8% for flesh color up to 22.4% for both tuber shape and eye depth. The strongest effect of year was found for tuber weight and it reached 14.5% of the variance in this trait.

**Table 2:** Analysis of variance in tuber shape, regularity of tuber shape, eye depth, tuber weight, and flesh color in the mapping population 11–36

Factor/interaction	*df*^a^ for effect	Mean Sum of squares effect	*F*	*p*	*R*^2^ (%)^b^
Tuber shape					
Genotype {G}	148.0	5.4	60.3	0.0000	72.6
Year {Y}	2.0	0.8	9.2	0.0001	0.2
G × Y	291.0	0.9	9.5	0.0000	22.4
Error	589.0	0.090			
Regularity					
Genotype {G}	148.0	1.2	8.4	0.0000	54.1
Year {Y}	2.0	0.4	3.0	0.0515	n.s.
G × Y	292.0	0.2	1.6	0.0000	20.4
Error	585.0	0.14			
Eye depth					
Genotype {G}	148.0	1.3	10.5	0.0000	55.0
Year {Y}	2.0	2.5	25.7	0.0000	1.8
G × Y	291.0	0.2	2.2	0.0000	22.4
Error	586.0	0.1			
Mean tuber weight					
Genotype {G}	148.0	792.0	18.1	0.0000	54.7
Year {Y}	2.0	15,578.0	356.1	0.0000	14.5
G × Y	291.0	138.0	3.2	0.0000	18.8
Error	587.0	44.0			
Tuber flesh color					
Genotype {G}	148.0	6.6	11.9	0.0000	60.4
Year {Y}	2.0	25.4	45.7	0.0000	3.1
G × Y	291.0	0.9	1.7	0.0000	16.4
Error	586.0	0.55			

*n.s.* not significant

^a^Number of degrees of freedom

^b^Percent of variance explained

The heritabilities of tuber shape, regularity, eye depth, tuber weight, and flesh color were *H*^2^ = 0.64, 0.59, 0.56, 0.61, and 0.67, respectively.

Several significant correlations were detected between analyzed traits (Table 3). Shallow eyes were strongly correlated with higher regularity. Round tubers tended to be more regular and had deeper yellow flesh. Tubers with lower weight had shallower eyes and were more regular than tubers with higher weight.

**Table 3:** Correlation coefficients (Pearson’s *r* values) computed on mean values over years between tuber shape, regularity of tuber shape, eye depth, tuber weight, and flesh color in mapping population 11–36

	Flesh color	Tuber shape	Regularity	Eye depth
Flesh color				
Tuber shape	− 0.16*			
Regularity	0.37**	− 0.17*		
Eye depth	0.30**	n.s.	0.77**	
Tuber weight	n.s.	n.s.	− 0.24*	− 0.39**

*n.s.* not significant

*Significant at α < 0.05

**Significant at α < 0.0001

### Linkage map

The final genetic map consisted of 1359 DArT markers and 9 SCAR and CAPS markers. A total length of the genetic map reached 972 cM. The lengths of the chromosomes ranged from 54 (chromosome IV) to 109 cM (chromosome I). The numbers of markers on particular chromosomes varied from 48 (chromosome IV) to 167 (chromosome II). Detailed description of genetic map was presented in Hara-Skrzypiec et al. (2017).

### QTL analysis

#### QTL for tuber shape

QTL for tuber shape were detected on four chromosomes: I, II, IV, and X. The most significant QTL for the trait was detected on chromosome X in four datasets (mean trait value and values from particular, testing years TS12–TS14). It explained up to 29.9% of the variance in mean dataset and was descending from DG 06-5 (Table 4). QTL for tuber shape on chromosomes I, II, and IV were significant for mean tuber shape and for 2 out of 3 years (Table 4 and Supplementary Table S1).

**Table 4:** QTL detected for mean (2012–2014) tuber shape, mean regularity of tuber shape, mean tuber weight, mean eye depth, and tuber flesh color in the mapping population 11–36. Only QTL with LOD > 3.0 are presented

Trait	Chromosome	Marker at peak or markers flanking map interval	Marker origin	Peak position (cM)	LOD	*R2* (%)	Interval (cM)
Tuber shape	I	pPt-472129	H	51.5	4.16	12.1	49.9–58.4
	II	pPt-538564	H	37.2	4.55	13.0	30.1–38.5
	IV	pPt-471810	P1	6.3	4.48	12.9	0.0–16.6
	X	pPt-559534	P2	40.5	11.50	29.9	23.7–64.4
Regularity of tuber shape	I	pPt-537507–pPt-471438	H	60.4	4.12	12.0	47.9–74.3
	III	pPt-538540	P2	25.9	5.49	15.6	17.9–38.5
	IV	pPt-651535	P1	22.9	3.91	11.4	21.4–30.6
	V	pPt-472014–pPt-655594	H, P1	45.1	3.87	11.3	43.2–51.7
	VIII	pPt-653841	P1	41.4	3.85	11.2	35.3–53.0
Eye depth	I	pPt-655486	P2	69.0	3.74	10.9	67.4–74.3
	III	pPt-459017	H	19.9	4.95	14.2	4.2–38.2
	IV	pPt-538354	P2	30.6	8.29	22.6	0.0–33.6
	V	pPt-650647	H	25.9	3.79	11.1	22.2–27.8
	V	pPt-655594	H	46.0	3.52	10.3	45.1–55.7
	XI	pPt-540308	H	44.8	5.06	14.5	44.0–45.5
Mean tuber weight	I	pPt-457903	P2	68.9	6.97	19.4	36.2–91.8
	IV	pPt-651535	P1	22.9	4.74	13.6	18.4–31.6
	V	pPt-654987	P1	32.5	3.74	10.9	23.4–36.8
	VI	pPt-654965	P2	41.2	5.52	15.7	22.8–66.2
Tuber flesh color	II	toPt-438004	P2	76.8	3.64	10.6	76.2–77.5
	III	TFC	P1	38.5	47.22	76.8	0.0–54.9

*P1* inherited from DG 03-226, *P2* inherited from DG 06-5, *H* descended from both parent

#### QTL for regularity of tuber shape

**Fig. 2:**
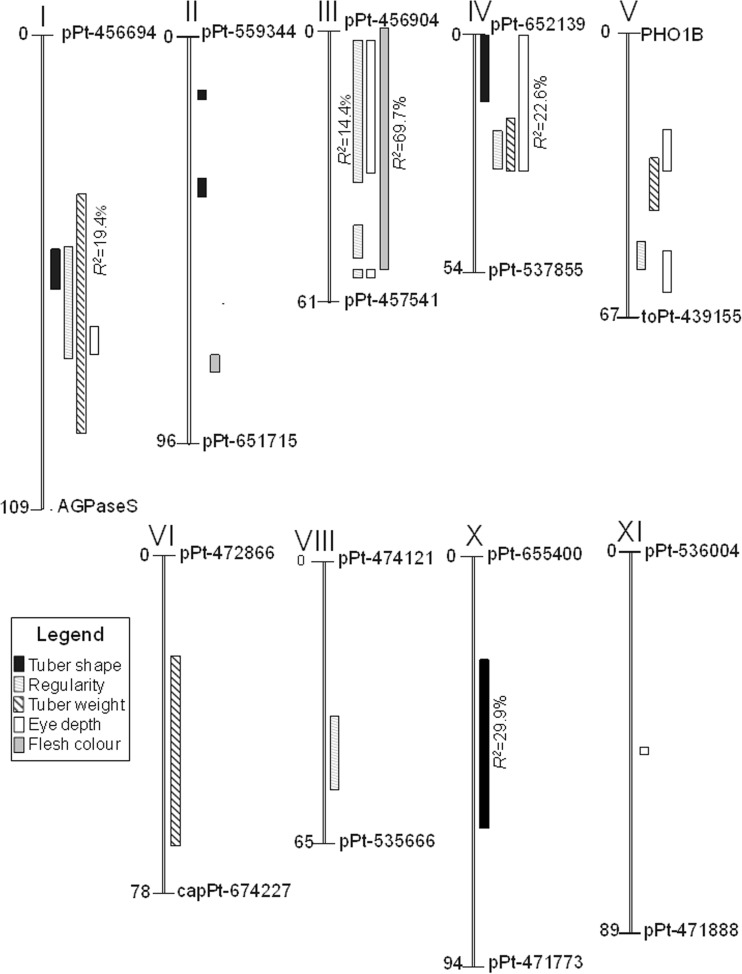
QTL on map of population 11–36 for tuber shape, regularity of tuber shape, mean eye depth, tuber weight, and tuber flesh color. QTL with the highest values of *R*^2^ for each analyzed trait are shown

Seven QTL for regularity of tuber shape were detected. Single QTL were detected on each of chromosomes I, IV, V, and VIII, while on chromosome III, up to three QTL might be present (Fig. 2). The strongest QTL for tuber regularity was identified on chromosome III and explained 15.6% of trait variance in mean dataset (Table 4). It was significant for four datasets and descended from both parents. QTL on chromosomes I, III, and IV were significant for mean regularity and for datasets from 2013 to 2014, while QTL on chromosomes V and VIII were significant for mean trait value and for datasets from single year of testing 2012 or 2013 (Table 4 and Supplementary Table S1).

#### QTL for eye depth

Seven QTL for eye depth were detected on five chromosomes: I, III (two QTL), IV, V (two QTL), and XI (Fig. 2). The most important QTL for the trait was detected on chromosome IV. This QTL explained 22.6% of variance in mean dataset (Table 4) and was inherited from DG 06-5. The QTL on chromosomes I, III, VII, and X were significant for mean eye depth scores and for one or two datasets out of three datasets for particular years: 2012–2014 (Table 4 and Supplementary Table S1).

#### QTL for tuber weight

QTL for tuber weight were detected on chromosomes I, IV, V, and VI (Fig. 2). The most important QTL was identified on chromosome I and was significant in all four datasets (mean value of trait and all datasets for particular years). It explained 19.4% of tuber weight variance in mean dataset and descended from DG 06-5 (Table 4). Other QTL were significant for two from three datasets from particular years (Table 4 and Supplementary Table S1).

#### QTL for tuber flesh color

The most important QTL for tuber flesh color was detected on chromosome III (Fig. 2). The effect of this QTL (LOD = 38.63) explained 76.8% of the variance for mean flesh color, and it descended from both parents (Table 4). Another QTL for flesh color, significant for mean trait value and for datasets from 2012 and 2014, was localized on chromosome II. It descended from DG 0–5 (Table 4 and Supplementary Table S1).

QTL intervals for tuber shape, regularity, tuber weight, and eye depth partially overlapped on chromosome I. On chromosome III, overlapping QTL were detected for regularity, eye depth, and tuber flesh color. QTL for tuber shape and eye depth but also for the regularity, eye depth, and tuber weight overlapped on chromosome IV, while QTL for eye depth partially overlapped with QTL for tuber weight and for regularity of tuber shape on chromosome V. In some cases, relationships between traits were confirmed by significant correlations between phenotypic data (Table 3).

### Discussion

For the first time, the QTL analysis of important morphological tuber traits was based on thorough phenotypic evaluation of tubers from a 3-year field experiment. So far, the published results were based on 1- or 2-year experiments (Li et al. 2005; Śliwka et al. 2008; Prashar et al. 2014; Manrique-Carpintero et al. 2015). Correlation coefficients between years, broad-sense heritabilities, and variance analysis (Table 2) showed that genetic factors are the most important in determination of all analyzed traits. The 3-year evaluation was important for determining significance of genotype × year interactions in the studied traits. The effects of these interactions were highly significant and only from 2.4 to 3.6 times smaller than variation determined by genotypes for respective traits. Therefore, 3-year phenotyping ensured that further analysis detected QTL accurately and estimated their effects more precisely that in studies based on single year experiment. The strongest QTL detected in our study were significant in all four datasets (3 years of testing and the mean), while other QTL were significant only in 1–3 datasets emphasizing the effect of the testing environments especially pronounced in case of the weaker QTL.

The variability in the eye depth and the regularity of tuber shape in our mapping population was narrowed, which is expected for the advanced breeding materials. However, it made our material less suitable for studying the inheritance of these traits and might have affected the heritabilities and QTL identified for the eye depth and the regularity of tuber shape.

Broad-sense heritability for tuber shape was 0.64 and for eye depth 0.56. In other studies estimated broad-sense heritability for tuber shape was in range 0.80–0.90 (Van Eck et al. 1994; Prashar et al. 2014) and for eye depth 0.82 (Prashar et al. 2014). In our study, heritability for regularity of tuber shape was moderate (0.58), while in Bradshaw et al. (2008), heritability of trait was estimated as high (0.845). In our study, heritability for tuber weight was 0.61. In other studies heritability of this trait was between 0.28 (Manrique-Carpintero et al. 2015) to 0.87 (Bradshaw et al. 2008). One of the possible sources of differences between broad-sense heritability values obtained in various studies could be different genetic composition of used plant material. Another explanation of observed differences could be the occurrence of smaller or larger differences between year and environmental effects in cited studies. The differences in heritabilities could have resulted also from different heritability calculation methods used. In our study, heritability was calculated as the ratio of genetic to total variance and differed from the heritability of progeny (clone) means averaged over years and replicates.

Although, the continuous variation from round to long tuber shape suggests a polygenic inheritance of the trait, early studies of tuber shape genetics described it as a monogenic trait (Taylor 1978; Okwuagwu 1981; Masson 1985). In some studies, there was evidence of the existence of minor loci affecting tuber shape but the trait has been reported as encoded by a major locus *Ro* on chromosome X (van Eck et al. 1994). This locus was closely linked with a major locus for eye depth at a distance of about 4 cM (Li et al. 2005). Van Eck (2007) hypothesized on pleiotropic effect of one locus on both traits or two closely linked loci influencing eye depth and tuber shape. In studies of Lindqvist-Kreuze et al. (2015), the QTL for eye depth and QTL for general tuber shape overlap on chromosome X with additional QTL for tuber shape found on chromosome V and QTL for eye depth on chromosome XII. Our results led to the conclusion that tuber shape and eye depth could not be explained by a simple one or two loci model as shown in studies mentioned above. We did not find a correlation between the 3-year mean scores of tuber shape and eye depth. A weak correlation between traits was only found in 1 year of testing (2013, data not shown). Weak correlation between traits has also been found by Śliwka et al. (2008). The major QTL for tuber shape detected in our study on chromosome X was perhaps corresponding to the locus *Ro* but the dominant allele encoding round tubers was absent in our mapping population resulting from a cross of parents with round-oval and oval tubers. The QTL for tuber shape on chromosome X in our study did not co-localize with QTL for eye depth. Only minor QTL for tuber shape co-localize with the major QTL for eye depth on chromosome IV. Our conclusions on complex genetic architecture of these two traits are in agreement with results of Sørensen (2006), Śliwka et al. (2008), D’hoop et al. (2014), and Prashar et al. (2014). QTL for tuber shape on chromosome X and II were detected in our study at similar positions to those identified by Prashar et al. (2014). Identified by these authors, the most significant marker c1_8020 underlying QTL for tuber shape on chromosome X maps to the genome superscaffold PGSC0003DMB000000385 (Sharma et al. 2013). In our study DArT marker pPt-55934 explaining 29.9% of tuber shape variance, significant in all testing years, maps to genome superscaffold PGSC0003DMB000000446. These superscaffolds are separated only by a superscaffold PGSC0003DMB000000546 in the genome DM1–3. The most significant SNPs underlying QTL effect for tuber shape on chromosome II detected by Prashar et al. (2014), namely c1_5091 and c2_51115 depending on testing year, map to superscaffold PGSC0003DMB000000141. This superscaffold also contains the most significant markers StPAD4 and Gp321 detected in QTL for tuber shape by Śliwka et al. (2008). Another significant marker (GP86) detected by the same authors appears to be contained in superscaffold PGSC0003DMB000000406, adjacent to PGSC0003DMB000000141. The most significant marker underlying QTL, detected in our study, on chromosome II: pPt-538564 maps to superscaffold PGSC0003DMB000000265. This superscaffold is separated from PGSC0003DMB000000141 by two superscaffolds, PGSC0003DMB000000552 and PGSC0003DMB000000204. Additionally, we detected minor QTL for the trait on chromosomes I and IV. QTL identified by us on chromosome I was also detected by Prashar et al. (2014), but in different position. We did not find QTL for tuber shape on chromosomes V, VI, VII, IX, XI, and XII identified by other authors (Sørensen 2006; Śliwka et al. 2008; D’hoop et al. 2014 and Prashar et al. 2014).

The regularity of tuber shape is a complex trait, in which depth of tuber eyes is an important component of the evaluation. A strong relationship between these traits was confirmed by correlation and QTL analyses. QTL for these traits colocalized on chromosomes I, III, IV, and V. Additionally, we detected QTL specific for each trait: for regularity on chromosome VIII and for eye depth on chromosome XI. QTL for regularity detected by Śliwka et al. (2008) on chromosome III was in similar position as QTL identified in our study. QTL on chromosome V detected by us was probably in a different position than the QTL detected by Śliwka et al. (2008). We did not detect QTL for trait on chromosome XI described by Śliwka et al. (2008). QTL analysis for regularity of tuber shape performed on a tetraploid full-sib family conducted by Bradshaw et al. (2008) enabled detection of three QTL for trait on chromosomes V, II, and XI. The QTL detected by us on chromosome V was in similar position as QTL detected by Bradshaw et al. (2008).

We detected a major QTL for eye depth on chromosome IV explaining 22.6% of trait variance and minor QTL on chromosomes I, III, V, and XI. We did not find QTL for eye depth on chromosome X and II identified by Śliwka et al. (2008) and Prashar et al. (2014). Additionally, in our study, we detected QTL for eye depth on chromosome III. Markers flanking one of two QTL on chromosome III, pPt-650377 and pPt-471937, map to superscaffold PGSC0003DMB000000026. It is adjacent to superscaffold PGSC0003DMB000000040 in which marker c2_37119, identified by Prashar et al. (2014), lying within QTL for eye depth on chromosome III, is mapped. It is very likely that the QTL for eye depth identified by us on chromosome V is in the corresponding position to QTL detected by Śliwka et al. (2008).

QTL detected for tuber weight in our study were located on chromosomes I, IV, V, and VI as in studies of Manrique-Carpintero et al. (2015). The most significant QTL for tuber weight detected by us on chromosome I (position on the DM1–3 physical map: chr01:70410943..70411368–chr01:87569573..87570249) included marker c2_12126 which was found by Manrique-Carpintero et al. (2015) at peak position of QTL on chromosome I. Our QTL on chromosomes IV, V, and VI are probably in different positions to QTL detected by Manrique-Carpintero et al. (2015). Size of tubers was analyzed in studies of Bradshaw et al. (2008) and D’hoop et al. (2014). QTL for this trait were detected on chromosomes V (Bradshaw et al. 2008), VIII, and IV (D’hoop et al. 2014). The position of QTL on chromosome IV was similar in our study and in work of D’hoop et al. (2014).

We detected a major QTL for flesh color on chromosome III that explained 76.8% of the trait variance (LOD = 38.63). A direct effect of this major QTL is visible in the bimodal distribution of the trait. Additionally, a minor QTL for the trait was localized on chromosome II. Our results confirmed the importance of locus/loci on chromosome III in determination of the trait which has been reported by Bonierbale et al. (1988), Brown et al. (2006), and Kloosterman et al. (2010). So far, *phytoene synthase* and *beta-carotene hydroxylase* involved in the carotenoid pathway have been found as potential candidate genes controlling yellow flesh color (Thorup et al. 2000; Brown et al. 2006; Kloosterman et al. 2010). Brown et al. (2006) have indicated that dominant allele B of gene *bch* is required for conferring yellow tuber flesh in potato. The variation in carotenoid content between BB and Bb genotypes suggests contributions of other genetic factors in total carotenoid level. Results of Kloosterman et al. (2010) studies have indicated that dominant allele B of *bch* appears to act through enhanced expression level. According to these authors, variation of *bch* expression derives from a polymorphism located physically near the gene. It was proven by identification of a large eQTL on the genetic position of *bch* (Kloosterman et al. 2010). The most significant marker pPt-535581 underlying QTL for flesh color on chromosome III was detected by us at a similar position to *bch.* This marker is mapped in superscaffold PGSC0003DMB000000127. It is separated by PGSC0003DMB000000400 from PGSC0003DMB000000159, in which *beta-carotene hydroxylase* (PGSC0003DMG400010169) is located. Similarly to our results, study of Śliwka et al. (2008) has shown that flesh color is not only determined by one locus. It is likely that QTL for flesh color on chromosome II detected in our study may involve genetic factors regulating carotenoid synthesis pathway. One possible candidate gene could be the gene encoding *zeaxanthin epoxidase* which maps to superscaffold PGSC0003DMB000000012 in the DM1–3 genome. The most significant marker underlying QTL for flesh color on chromosome II (pPt-438004) maps to superscaffold PGSC0003DMB000000441. These two superscaffolds are separated only by one small superscaffold PGSC0003DMB000001213.

The identification of many QTL within the potato genome influencing tuber shape, regularity of tuber shape, eye depth, and mean tuber weight led to conclusion on complex genetic architecture of these traits. Our analysis confirmed the importance of some QTL detected in previous studies, and these QTL can be regarded as universal, i.e., present in diversified materials studied so far and effective regardless of the genetic background. We also identified new loci affecting the variation in studied traits. Detection of these QTL could be a result of using a mapping population having a number of wild introgressions. It is likely that they could be a source of new alleles, which were not detected in populations used in other studies. An important achievement of our study is identification, besides a major QTL for tuber flesh color, of an additional QTL on chromosome II, which may be important in modification of tuber flesh color.

## Electronic supplementary material

ESM 1 (DOCX 18 kb)

## References

[CR1] Bonierbale MW, Plaisted RL, Tanksley SD (1988). RFLP maps based on common set of clones reveal modes of chromosomal evolution in potato and tomato. Genetics.

[CR2] Bradshaw JE, Hackett CA, Pande B, Waugh R, Bryan GJ (2008). QTL mapping of yield, agronomic and quality traits in tetraploid potato (*Solanum tuberosum* subsp. *tuberosum*). Theor Appl Genet.

[CR3] Brown CR, Edwards CG, Yang C-P, Dean BB (1993). Orange flesh trait in potato: inheritance and carotenoid content. J Am Soc Hortic Sci.

[CR4] Brown CR, Kim TS, Ganga Z, Haynes K, De Jong D, Jahn M, Paran I, De Jong W (2006) Segregation of total carotenoid in high level potato germplasm and its relationship to beta-carotene hydroxylase polymorphism. American Journal of Potato Research 83(5):365–372

[CR5] D’hoop BB, Keizer PL, Paulo MJ, Visser RG, van Euwijk FA, van Eck HJ (2014). Identification of agronomically important QTL in tetraploid potato cultivars using a marker-trait association analysis. Theor Appl Genet.

[CR6] Domański L (2001). Assessment of morphological characters of potato tubers. Monografie i Rozprawy Naukowe IHAR, Radzików. Poland.

[CR7] Domański L, Michalak K, Zimnoch – Guzowska E (2007) Variation of blackspot susceptibility of the selected potato cultivars. Biuletyn IHAR 246:145–149

[CR8] Hara-Skrzypiec A, Śliwka J, Jakuczun H, Zimnoch-Guzowska E (2017) Quantitative trait loci for tuber blackspot bruise and enzymatic discoloration susceptibility in diploid potato. Mol Gen Genomics. 10.1007/s00438-017-1387-0 10.1007/s00438-017-1387-0 PMC5854731 29080143

[CR9] Kloosterman B, Oortwijn M, Willigen JU, America T, de Vos R, Visser RGF, Bachem CWB (2010). From QTL to candidate gene: genetical genomics of simple and complex traits in potato using a pooling strategy. BMC Genomics.

[CR10] Li X-Q, De Jong H, De Jong DM, De Jong WS (2005). Inheritance and genetic mapping of tuber eye depth in cultivated diploid potatoes. Theor Appl Genet.

[CR11] Lindqvist-Kreuze H, Khan A, Salas E, Meiyalaghan S, Thompson S, Gomez R, Bonierbale M (2015). Tuber shape and eye depth variation in a diploid family of Andean potatoes. BMC Genet.

[CR12] Manrique-Carpintero NC, Coombs JJ, Cui Y, Veillux RE, Buell CR, Douches D (2015). Genetic map and QTL analysis of agronomic traits in diploid potato population using single nucleotide polymorphism markers. Crop Sci.

[CR13] Masson MF (1985) Mapping, combining abilities, heritabilities and heterosis with 4x × 2x crosses in potato. PH.D. Thesis, University of Wisconsin, Madison

[CR14] Morris WL, Ducreux GDW, Stewart D, Davies HV, Taylor MA (2004). Carotenogenesis during tuber development and storage in potato. J Exp Bot.

[CR15] Okwuagwu CO (1981) Phenotypic evaluation and cytological analysis of 24-chromosome hybrids for analytical breeding in potato. Ph.D. Thesis, University of Wisconsin, Madison

[CR16] Prashar A, Hornyik C, Young V, McLean K, Sharma SK, Dale MFB, Bryan GJ (2014). Construction of dense SNP map of highly heterozygous diploid potato population and QTL analysis of tuber shape and eye depth. Theor Appl Genet.

[CR17] Sharma SK, Bolser D, de Boer J, Sønderkær M, Amoros W, Carboni MF, D’Ambrosio JM, de la Cruz G, Di Genova A, Douches DS, Eguiluz M, Guo X, Guzman F, Hackett CA, Hamilton JP, Li G, Li Y, Lozano R, Maass A, Marshall D, Martinez D, McLean K, Mejía N, Milne L, Munive S, Nagy I, Ponce O, Ramirez M, Simon R, Thomson SJ, Torres Y, Waugh R, Zhang Z, Huang S, Visser RG, Bachem CW, Sagredo B, Feingold SE, Orjeda G, Veilleux RE, Bonierbale M, Jacobs JM, Milbourne D, Martin DM, Bryan GJ (2013). Construction of reference chromosome-scale pseudomolecules for potato: integrating the potato genome with genetic and physical maps. G3 Genes Genomes Genet.

[CR18] Śliwka J, Wasilewicz-Flis I, Jakuczun H, Gebhardt C (2008). Tagging quantitative trait loci for dormancy, tuber shape, regularity of tuber shape, eye depth and flesh colour in diploid potato originated from six *Solanum* species. Plant Breed.

[CR19] Śliwka J, Jakuczun H, Chmielarz M, Hara-Skrzypiec A, Tomczyńska I, Kilian A, Zimnoch-Guzowska E (2012). A new resistance gene against potato late blight originating from *Solanum × michoacanum* maps to potato chromosome VII. Theor Appl Genet.

[CR20] Śliwka J, Jakuczun H, Chmielarz M, Hara-Skrzypiec A, Tomczyńska I, Kilian A, Zimnoch-Guzowska E (2012). Late blight resistance gene from *Solanum ruiz-ceballosii* is located on potato chromosome X and linked to violet flower colour. BMC Genet.

[CR21] Sørensen KK (2006)*:* Mapping of morphological traits and associations with late blight resistance in *Solanum tuberosum* and *S. vernei. In:* QTLs for foliage late blight resistance from *Solanum vernei*, 93*—*122*.* PhD thesis*.* Danish Institute of Agricultural Sciences, Royal Veterinary and Agricultural University, Frederiksberg, Denmark

[CR22] Taylor LM (1978). Variation patterns of parthenogenetic plants derived from unreduced embryo-sac of *Solanum tuberosum* subspecies *andigena* (Juz.et Buk.) Hawkes. Theor Appl Genet.

[CR23] Thorup TA, Tanyolac B, Livingstone KD, Popovsky S, Paran I, Jahn M (2000). Candidate gene analysis of organ pigmentation loci in the Solanaceae. Proc Natl Acad Sci U S A.

[CR24] Van Eck HJ (2007) Genetics of morphological and tuber traits in potato biology and biotechnology advances and perspectives. In: Dick Vreugdenhil (ed) Elsevier Oxford, Amsterdam pp 91–115. http://solanaceae.plantbiology.msu.edu/integrated_searches.shtml

[CR25] Van Eck HJ, Jacobs JME, Stam P, Ton J, Stiekema WJ, Jacobsen E (1994). Multiple alleles for tuber shape in diploid potato detected by qualitative and quantitative genetic analysis using RFLPs. Genetics.

[CR26] Van Ooijen JW (2006). JoinMap ® 4, software for the calculation of the genetic linkage maps in experimental populations.

[CR27] Van Ooijen JW (2009). MapQTL ® 6, software for mapping of quantitative trait loci in experimental populations of diploid species.

[CR28] Wolters A-MA, Uitdewilligen JGAML, Kloosterman BA, Hutten RCB, Visser RGF, van Eck HJ (2010). Identification of alleles of carotenoid pathway genes important for zeaxanthin accumulation in potato tubers. Plant Mol Biol.

