# Supplementary material for: QTL for tuber morphology traits in diploid potato
Source: J Appl Genet. 2018 Feb 28;59(2):123–32. doi: 10.1007/s13353-018-0433-x (PMC5895667; doi:10.1007/s13353-018-0433-x)
Supplement: Supplementary file 1 — (DOCX 18 kb) [file 13353_2018_433_MOESM1_ESM.docx]

Supplementary Table S1. QTL detected in particular years of phenotyping in the mapping population 11-36. Only QTL with LOD>3.0 are presented.

| Trait | Chromosome | Marker at peak or markers flanking virtual interval | Marker origin ^a/^ | Peak position (cM) | LOD | *R2* (%) |
| --- | --- | --- | --- | --- | --- | --- |
| TS12 | I | pPt-472129 | H | 51.5 | 3.55 | 10.4 |
| TS13 |  |  |  |  | 4.15 | 12.0 |
| TS12 | II | pPt-538564 | H | 37.2 | 3.05 | 12.0 |
| TS14 |  |  |  |  | 4.34 | 13.0 |
| TS13 | IV | pPt-471810 | P1 | 6.3 | 3.65 | 10.7 |
| TS14 |  |  |  |  | 3.42 | 10.4 |
| **TS12** | **X** | **pPt-559534** | **P2** | **40.5** | **5.48** | **15.6** |
| **TS13** |  |  |  |  | **8.69** | **23.5** |
| **TS14** |  |  |  |  | **10.50** | **28.5** |
| ED12 | I | pPt-655486 | P2 | 69.0 | 4.93 | 14.1 |
| ED12 | I | pPt-652203 | P1 | 78.8 | 4.60 | 13.3 |
| ED12 | III | pPt-459017 | H | 19.9 | 4.04 | 11.7 |
| ED14 |  |  |  |  | 4.52 | 13.5 |
| **ED12** | **IV** | **pPt-538354** | **P2** | **30.6** | **5.55** | **15.8** |
| **ED13** |  |  |  |  | **7.23** | **20.0** |
| **ED14** |  |  |  |  | **4.51** | **13.4** |
| ED12 | V | pPt-650647 | H | 25.9 | 3.08 | 9.1 |
| ED13 |  |  |  |  | 3.98 | 11.6 |
| ED13 | V | pPt-655594 | H | 46.0 | 3.89 | 11.3 |
| ED13 | XI | pPt-540308 | H | 44.8 | 4.91 | 14.1 |
| ED14 |  |  |  |  | 3.19 | 9.7 |
| REG13 | I | pPt-537507- pPt-471438 | H | 60.4 | 4.05 | 11.8 |
| REG14 |  |  |  |  | 3.04 | 9.3 |
| **REG12** | **III** | **pPt-538540** | **P2** | **25.9** | **4.47** | **12.9** |
| **REG13** |  |  |  |  | **4.95** | **14.2** |
| **REG14** |  |  |  |  | **3.57** | **10.8** |
| REG13 | IV | pPt-651535 | P1 | 22.9 | 3.12 | 9.2 |
| REG14 |  |  |  |  | 5.30 | 15.6 |
| REG12 | V | pPt-472014- pPt-655594 | H, P1 | 45.1 | 4.87 | 14.0 |
| REG13 | VIII | pPt-653841 | P1 | 41.4 | 4.84 | 13.9 |
| **MTW12** | **I** | **pPt-457903** | **P2** | **68.9** | **5.08** | **14.5** |
| **MTW13** |  |  |  |  | **3.52** | **10.3** |
| **MTW14** |  |  |  |  | **6.71** | **19.3** |
| MTW13 | IV | pPt-651535 | P1 | 22.9 | 5.00 | 14.3 |
| MTW14 |  |  |  |  | 4.35 | 13.0 |
| MTW12 | V | pPt-654987 | P1 | 32.5 | 3.94 | 11.5 |
| MTW13 |  |  |  |  | 4.70 | 13.5 |
| MTW12 | VI | pPt-654965 | P2 | 41.2 | 3.62 | 10.6 |
| MTW14 |  |  |  |  | 6.18 | 17.9 |
| TFC12 | II | toPt-438004 | P2 | 76.8 | 3.32 | 9.7 |
| TFC14 |  |  |  |  | 3.92 | 11.8 |
| **TFC12** | **III** | **TFC** | **P1** | **38.5** | **38.02** | **69.1** |
| **TFC13** |  |  |  |  | **33.21** | **64.2** |
| **TFC14** |  |  |  | **25.22** | **21.51** | **47.2** |

^a/^ P1- inherited from DG 03-226, P2- inherited from DG 06-5, H- descended from both parents

TS - tuber shape, REG - regularity of tuber shape, MTW - mean tuber weight, ED- eye depth, TFC – tuber flesh colour.
